# Personal

**Published:** 1898-05

**Authors:** 


### PERSONAL.

DR. JAMES W. MCLANE, who for thirty years has been Professor of Obstetrics at the College of Physicians and Surgeons, the medical department of Columbia University, has resigned his teaching chair, but retains his place as dean of the medical faculty. Dr. McLane is the type of men that not only ornament the medical profession but who increase confidence in the guild. The medical department of Columbia can ill-afford to part with him. The vacancy thus created has been filled by the appointment of Dr. Edwin B. Cragin as lecturer in obstetrics. Dr. Cragin is a graduate of Yale and of the College of Physicians and Surgeons, and in his positions at the Roosevelt and Cancer Hospitals he has manifested conspicuous ability. In his new position he will have professional charge of the Sloane Maternity Hospital, connected with the College of Physicians and Surgeons, and he will also have entire direction of the obstetrical work of the students.

DR. ROLLIN T. ROLPH, of Dunkirk, recently appointed health officer, has, in compliance with the law, resigned as a member of the Board of Health. Dr. Rolph has also been appointed one of the non-resident staff of the Buffalo Hospital of the Sisters of Charity.

Dr. W. Scott Renner, of Buffalo, Professor of Laryngology at Niagara University, sailed on Saturday, April 23, 1898, for a three months' tour in Europe. He was accompanied by Mrs. Renner and her father and mother, Mr. and Mrs. Diehl.

---

Dr. E. L. Ruffner and Mrs. Jennie L. Thomas, of Buffalo, were married April 18, 1898. Dr. Ruffner has removed his office from 282 Hampshire street to 181 Allen street. Hours: 8–10 A. M., 2–3 and 7–8 P. M.

---

Dr. C. F. Durrand, of Buffalo, has removed from 258 Franklin street to room 403 Mooney-Brisbane building. Dr. Durrand has been appointed a surgeon on the staff of the German Hospital.

---

Dr. Edgar A. Forsyth, of Buffalo, who limits his practice to diseases of the nose, throat and ear, has removed from 64 West Huron street to 348 Franklin street. Hours: 9–1 and 7–8.

---

Dr. J. J. Finerty, of Buffalo, whose practice is limited to diseases of the eye and ear, has removed from 266 to 429 Franklin street. Hours: 9–1. Telephone.

---

Dr. J. Henry Dowd, of Buffalo, has removed his office from 206 to 223 Franklin street. Hours: 9–1 and 7–8; Sundays, 2–4 P. M.

---

Dr. L. G. Hanley, of Buffalo, has established an office at 74 Franklin street. Hours: 1–4 P. M.

---

Dr. George S. Palmer, of Buffalo, has removed from 316 to 280 Franklin street.

